# Supplementary material for: Different clinical impact of hyperuricemia according to etiologies of chronic kidney disease: Gonryo Study
Source: PLoS One. 2021 Mar 25;16(3):e0249240. doi: 10.1371/journal.pone.0249240 (PMC7993817; doi:10.1371/journal.pone.0249240)
Supplement: S1 Table — (DOCX) [file pone.0249240.s001.docx]

**S1 Table. Associations of uric acid with renal and non-renal outcomes.**

|  | **Univariate** | |  | **Multivariate†** | |
| --- | --- | --- | --- | --- | --- |
|  | **HR (95% CI)** | ***P* value** |  | **HR (95% CI)** | ***P* value** |
| Renal outcome (ESRD) |  |  |  |  |  |
| Overall | 1.541 (1.464 – 1.623) | <0.001 |  | 1.034 (0.962 – 1.111) | 0.366 |
| Male | 1.514 (1.409 – 1.626) | <0.001 |  | 1.105 (1.011 – 1.207) | 0.027 |
| Female | 1.554 (1.438 – 1.680) | <0.001 |  | 0.911 (0.795 – 1.043) | 0.177 |
| PRD | 1.639 (1.471 – 1.825) | <0.001 |  | 0.940 (0.815 – 1.085) | 0.399 |
| HTN | 1.471 (1.295 – 1.670) | <0.001 |  | 1.108 (0.920 – 1.333) | 0.280 |
| DN | 1.309 (1.204 – 1.423) | <0.001 |  | 1.039 (0.896 – 1.204) | 0.615 |
| Others | 1.531 (1.375 – 1.705) | <0.001 |  | 1.022 (0.865 – 1.206) | 0.802 |
| Deaths |  |  |  |  |  |
| Overall | 1.300 (1.178 – 1.434) | <0.001 |  | 0.996 (0.876 – 1.133) | 0.953 |
| Male | 1.084 (0.939 – 1.251) | 0.273 |  | 0.960 (0.824 – 1.117) | 0.595 |
| Female | 1.529 (1.327 – 1.761) | <0.001 |  | 1.137 (0.912 – 1.418) | 0.254 |
| PRD | 1.377 (1.106 – 1.714) | 0.004 |  | 1.266 (0.952 – 1.682) | 0.104 |
| HTN | 1.364 (1.137 – 1.637) | <0.001 |  | 1.534 (1.134 – 2.073) | 0.005 |
| DN | 1.108 (0.913 – 1.345) | 0.300 |  | 0.557 (0.376 – 0.826) | 0.004 |
| Others | 1.243 (1.038 – 1.488) | 0.018 |  | 0.925 (0.679 – 1.260) | 0.622 |
| CVEs |  |  |  |  |  |
| Overall | 1.203 (1.110 – 1.305) | <0.001 |  | 1.086 (0.978 – 1.207) | 0.122 |
| Male | 1.091 (0.966 – 1.232) | 0.161 |  | 1.051 (0.917 – 1.203) | 0.475 |
| Female | 1.299 (1.160 – 1.456) | <0.001 |  | 1.137 (0.962 – 1.343) | 0.131 |
| PRD | 1.385 (1.148 – 1.670) | 0.001 |  | 1.324 (1.024 – 1.711) | 0.032 |
| HTN | 1.085 (0.930 – 1.267) | 0.299 |  | 1.152 (0.926 – 1.434) | 0.205 |
| DN | 1.031 (0.891 – 1.192) | 0.686 |  | 0.919 (0.742 – 1.138) | 0.438 |
| Others | 1.238 (1.062 – 1.434) | 0.006 |  | 1.180 (0.953 – 1.461) | 0.129 |
| Non-renal outcome (CVEs and deaths) |  |  |  |  |  |
| Overall | 1.228 (1.146 – 1.315) | <0.001 |  | 1.050 (0.960 – 1.149) | 0.288 |
| Male | 1.095 (0.989 – 1.213) | 0.079 |  | 1.012 (0.906 – 1.132) | 0.828 |
| Female | 1.342 (1.216 – 1.480) | <0.001 |  | 1.140 (0.982 – 1.322) | 0.085 |
| PRD | 1.311 (1.116 – 1.539) | 0.001 |  | 1.248 (1.003 – 1.553) | 0.047 |
| HTN | 1.192 (1.045 – 1.359) | 0.009 |  | 1.253 (1.035 – 1.516) | 0.021 |
| DN | 1.044 (0.919 – 1.186) | 0.505 |  | 0.851 (0.706 – 1.026) | 0.851 |
| Others | 1.243 (1.097 – 1.408) | 0.001 |  | 1.066 (0.887 – 1.280) | 0.495 |

CI, confidence interval; HR, hazard ratio; PRD, primary renal disease; HTN, hypertensive nephropathy; DN, diabetic nephropathy; CVEs, cardiovascular events.

†Adjusted for underlying disease of CKD, eGFR, urinary protein, systolic blood pressure, past history of cardiac disease, ACEi/ARBs, statins, antiplatelets, diuretics, allopurinol, age, sex, smoking, body mass index, hemoglobin, albumin.
