# Supplementary material for: Different clinical impact of hyperuricemia according to etiologies of chronic kidney disease: Gonryo Study
Source: PLoS One. 2021 Mar 25;16(3):e0249240. doi: 10.1371/journal.pone.0249240 (PMC7993817; doi:10.1371/journal.pone.0249240)
Supplement: S2 Table — (DOCX) [file pone.0249240.s002.docx]

**S2 Table. Associations of allopurinol with renal and non-renal outcomes.**

|  | **Univariate** | |  | **Multivariate†** | |
| --- | --- | --- | --- | --- | --- |
|  | **HR (95% CI)** | ***P* value** |  | **HR (95% CI)** | ***P* value** |
| Renal death (ESRD) |  |  |  |  |  |
| Overall | 3.077 (2.502 – 3.785) | <0.001 |  | 1.021 (0.782 – 1.335) | 0.877 |
| Male | 2.398 (1.850 – 3.108) | <0.001 |  | 1.026 (0.735 – 1.430) | 0.881 |
| Female | 4.274 (2.963 – 6.165) | <0.001 |  | 0.916 (0.554 – 1.516) | 0.733 |
| PRD | 4.852 (3.341 – 7.045) | <0.001 |  | 0.830 (0.503 – 1.368) | 0.464 |
| HTN | 2.259 (1.337 – 3.815) | 0.002 |  | 0.609 (0.239 – 1.554) | 0.300 |
| DN | 2.529 (1.704 – 3.753) | <0.001 |  | 1.451 (0.898 – 2.345) | 0.129 |
| Others | 3.214 (2.101 – 4.918) | <0.001 |  | 1.040 (0.582 – 1.858) | 0.894 |
| Deaths |  |  |  |  |  |
| Overall | 1.313 (0.891 – 1.934) | 0.169 |  | 0.819 (0.490 – 1.369) | 0.447 |
| Male | 1.094 (0.700 – 1.710) | 0.692 |  | 0.943 (0.532 – 1.672) | 0.840 |
| Female | 1.103 (0.434 – 2.807) | 0.837 |  | 0.392 (0.100 - 1.540) | 0.180 |
| PRD | 1.314 (0.585 – 2.952) | 0.508 |  | 0.767 (0.265 – 2.217) | 0.624 |
| HTN | 0.678 (0.306 – 1.503) | 0.338 |  | 0.718 (0.240 – 2.150) | 0.554 |
| DN | 1.482 (0.586 – 3.745) | 0.406 |  | 0.307 (0.063 – 1.489) | 0.143 |
| Others | 1.922 (0.989 – 3.736) | 0.054 |  | 1.915 (0.756 – 4.850) | 0.170 |
| CV events |  |  |  |  |  |
| Overall | 1.336 (0.978 – 1.826) | 0.069 |  | 1.012 (0.666 – 1.537) | 0.956 |
| Male | 1.033 (0.704 – 1.518) | 0.867 |  | 1.029 (0.624 – 1.695) | 0.912 |
| Female | 1.937 (1.102 – 3.405) | 0.022 |  | 1.043 (0.489 – 2.317) | 0.918 |
| PRD | 1.683 (0.872 – 3.250) | 0.121 |  | 1.305 (0.522 – 3.262) | 0.568 |
| HTN | 0.784 (0.433 – 1.420) | 0.423 |  | 0.607 (0.251 – 1.467) | 0.267 |
| DN | 1.823 (0.946 – 3.513) | 0.073 |  | 1.798 (0.691 – 4.674) | 0.229 |
| Others | 1.304 (0.714 – 2.382) | 0.388 |  | 1.374 (0.584 – 3.233) | 0.466 |
| CV events and deaths |  |  |  |  |  |
| Overall | 1.288 (0.985 – 1.684) | 0.065 |  | 0.977 (0.685 – 1.392) | 0.897 |
| Male | 1.007 (0.730 – 1.389) | 0.967 |  | 1.033 (0.684 – 1.559) | 0.878 |
| Female | 1.666 (0.988 – 2.809) | 0.055 |  | 0.871 (0.414 – 1.831) | 0.716 |
| PRD | 1.675 (0.958 – 2.928) | 0.070 |  | 1.362 (0.639 – 2.903) | 0.424 |
| HTN | 0.607 (0.348 – 1.059) | 0.078 |  | 0.552 (0.258 – 1.181) | 0.126 |
| DN | 1.557 (0.859 – 2.823) | 0.144 |  | 1.002 (0.429 – 2.342) | 0.997 |
| Others | 1.593 (0.993 – 2.554) | 0.054 |  | 1.679 (0.873 – 3.228) | 0.120 |

CI, confidence interval; HR, hazard ratio; PRD, primary renal disease; HTN, hypertensive nephropathy; DN, diabetic nephropathy; CVEs, cardiovascular events.

†Adjusted for underlying disease of CKD, eGFR, urinary protein, systolic blood pressure, past history of cardiac disease, ACEi/ARBs, statins, antiplatelets, diuretics, uric acid, age, sex, smoking, body mass index, hemoglobin, albumi.
