# Supplementary material for: Different clinical impact of hyperuricemia according to etiologies of chronic kidney disease: Gonryo Study
Source: PLoS One. 2021 Mar 25;16(3):e0249240. doi: 10.1371/journal.pone.0249240 (PMC7993817; doi:10.1371/journal.pone.0249240)
Supplement: Attachment — Submitted filename: Response to Reviewers.docx [file pone.0249240.s003.docx]

***Additional Editor Comments:***

*The method to link follow-up outcomes should be described. Some important factors are missing in the adjusted model, such as Statin. Please add the potential confounders in the model or acknowledge them as a limitation.*

[Response]

National registry or database to link subjects’ outcomes do not exist in Japan. Nephrologists and their assistants in eleven participating hospitals in Miyagi Prefecture kept a record of each participants’ outcomes once a year in medical records and picked up them for analysis. Regarding this point, we added the following comment in Methods section.

Line 80-81, Nephrologists and their assistants in participating hospitals kept a record of each participants’ outcomes once a year in medical records and picked up them for analysis.

Following your comment, we re-analyzed the adjusted model adding statins and antiplatelets as the potential confounders. Please, see figures and tables (**Fig. 3** and **4**, **S1** and **S2 Table**). As the result of re-analysis, we identified primary renal disease (PRD) patients also have the risk for non-renal outcomes. Therefore, we modified manuscript as follows.

Line 40-43, but in primary renal disease (PRD) and hypertensive renal disease (HTN) patients, hyperuricemia was significantly associated with non-renal outcomes on multivariate Cox proportional analysis (adjusted hazard ratio [95% confidence interval] PRD, 1.248 [1.003 – 1.553]; HTN, 1.250 [1.035 – 1.510]).

Line 47, Hyperuricemia is an independent risk factor for non-renal outcomes in primary renal disease and hypertensive renal disease patients.

Line 172-173, In the subgroup analysis, ~~only~~ PRD and HTN was were associated with an increased risk for the non-renal outcome (~~HR 1.250, 95%CI 1.035-1.510, p=0.020~~ adjusted HR [95% Cl] PRD, 1.248 [1.003 – 1.553]; HTN, 1.250 [1.035 – 1.510]) (**Figure 3-b**).

Line 187-189, The results of this study demonstrated that hyperuricemia is an independent risk factor for non-renal outcomes in primary renal disease and hypertensive renal disease patients, and allopurinol did not decrease the risk in these patients.

Line 247-249, Hyperuricemia is an independent risk factor for non-renal outcomes in primary renal disease and hypertensive renal disease patients, and allopurinol did not decrease the risks for renal and non-renal outcomes.

Line 211-216, IgA nephropathy is the most common primary glomerulonephritis in Japan [29]. Although it has been suggested that IgA nephropathy patients with hyperuricemia have poor renal prognosis [12, 17], the relationship between hyperuricemia in IgA nephropathy patients and non-renal outcome was unclear. We confirmed that non-renal outcome in PRD is poor in this study, however, the data of detailed classification of PRD is lacking. It is necessary to clarify how the renal and non-renal outcomes differs depending on the type of PRD in the future.

[29] Sugiyama H, Yokoyama H, Sato H, Saito T, Kohda Y, Nishi S, et al. Japan Renal Biopsy Registry and Japan Kidney Disease Registry: Committee Report for 2009 and 2010. Clin Exp Nephrol 2013; 17(2): 155-73

***Reviewer #1:*** *Watanabe et al. investigated the effect of hyperuricemia on clinical outcomes in different CKD etiology. Hyperuricemia is an independent risk factor for non-renal outcomes in hypertensive renal disease. I have some comments to improve the study.*

*1. Primary renal disease was the major cause of CKD in the Gonryo study (2006 -2008). I wondered the proportion of major cause of CKD in Japan.*

[Response to 1]

Unfortunately, there are no Japanese patients’ data showing the proportion of major cause of CKD. Since the Gonryo study targets only CKD patients who had been followed by nephrologist, the proportion of nephritis patients is thought to be relatively high compared to the actual situation. The most common primary cause of renal failure among new dialysis patients was diabetic nephropathy (43.8%), followed by chronic glomerulonephritis (32.4%) in the nationwide survey conducted at the end of 2013 in Japan*. Based on the result, it is expected that there would actually be more DN/DKD patients compared to our result in **Table1**.

*Ikuto M, Shigeru N, Satoshi O, Naoki K, Norio H, Takayuki H, et al. An Overview of Regular Dialysis Treatment in Japan (As of 31 December 2013). Ther Apher Dial 2015; 19(6): 540-74

*2. The present in both figures and tables is redundant in the main text. Please move the detailed data of Table to the supplementary material.*

[Response to 2]

We moved the detailed data of Table (**Table 2** and **3**) to the supplementary material (**S1** and **S2 Table**).

*3. Page 15, line 203, the full name of “RCT”, “FEATHER”, “PERL”, and “CKD-FIX” should be presented.*

[Response to 3]

We corrected the sentence which you pointed out as follows.

Line 221-227, ~~However, recent RCTs, such as FEATHER, PERL, and CKD-FIX,~~ However, recent multicenter, randomized, double-blind, placebo-controlled trials (RCTs), such as “Febuxostat Versus Placebo Randomized Controlled Trial Regarding Reduced Renal Function in Patients With Hyperuricemia Complicated by Chronic Kidney Disease Stage 3” (FEATHER), the Preventing Early Renal Loss in Diabetes (PERL), and “the Controlled Trial of Slowing of Kidney Disease Progression from the Inhibition of Xanthine Oxidase” (CKD-FIX) failed to demonstrate clinically meaningful benefits of serum urate reduction with allopurinol or febuxostat on kidney function decline, similar to the present results [21][22][32].

*4. Since hyperuricemia is associated with non-renal outcomes in hypertensive renal disease patients, a spline function for uric acid level and non-renal outcomes will be interesting to observe the adequate and optimal uric acid cut off value.*

[Response to 4]

We detected that UA 6.0 mg/dL in PRD and UA 6.8 mg/dL in HTN are optimal cut off value from ROC analysis. Regarding this point, we added the following comment in Result section.

Line 174-176, UA 6.0 mg/dL in PRD and UA 6.8 mg/dL in HTN are optimal cut off value from ROC analysis. AUC of ROC curve and p-value are 0.611 (95%CI, 0.537-0.685, p=0.004) in PRD and 0.658 (95%CI, 0.584-0.732, p=0.012) in HTN.

*5. The observation subjects were enrolled between May 2006 and November 2008 and follow up until December 2015. How to link these participants and recorded their outcomes? Is there any national registry or database to link subjects’ outcomes?*

[Response to 5]

National registry or database to link subjects’ outcomes do not exist in Japan. Nephrologists and their assistants in eleven participating hospitals in Miyagi Prefecture kept a record of each participants’ outcomes once a year in medical records and picked up them for analysis. Regarding this point, we added the following comment in Methods section.

Line 80-81, Nephrologists and their assistants in participating hospitals kept a record of each participants’ outcomes once a year in medical records and picked up them for analysis.

***Reviewer #2:*** *The authors demonstrated the association between hyperuricemia and clinical outcomes in CKD patients in a prospective observational cohort study. It has novelty because the authors showed the effects of hyperuricemia varies according to underlying diseases causing CKD. However, there are some issues needed to be clarified.*

*Major issue:Q1: The authors should explain in more detail how to classify CKD patients into different categories, especially when patients had more than two underlying diseases causing CKD simultaneously. According to table 1, 149(31.9%) patients in hypertensive nephropathy group(HTN) had diabetes mellitus, and 249(90.5%) patients in diabetic nephropathy group(DN) had hypertension. However, only 12.8% and 17.1% patients in HTN and DN respectively received renal biopsy. Without biopsy, how did the authors judge and confirm the underlying diseases causing CKD?*

[Response to Q1]

Patients were classified according to one of four underlying renal diseases diagnosed by the attending physicians at the participating hospitals. Hypertensive nephropathy was defined by preceding history of hypertension with absence of other possible disorders, including cases with biopsy findings of nephrosclerosis, and diabetic nephropathy was defined by preceding history of diabetes accompanying nephropathy with absence of other possible renal disorders, including cases with biopsy findings of diabetic nephropathy or those who presenting nephropathy with diabetic retinopathy in the absence of other possible disorders. Following your suggestion, we added the detailed explanation in the methods section.

Line 95-100, Hypertensive nephropathy was defined by preceding history of hypertension with absence of other possible disorders, including cases with biopsy findings of nephrosclerosis, and diabetic nephropathy was defined by preceding history of diabetes accompanying nephropathy with absence of other possible renal disorders, including cases with biopsy findings of diabetic nephropathy or those who presenting nephropathy with diabetic retinopathy in the absence of other possible disorders.

*Q2: Statins have been proven to be effective in the primary and secondary prevention of cardiovascular disease. However, statins were not adjusted in the evaluation of non-renal outcomes (CVEs and deaths) in the present study. Meanwhile, information about the use of antiplatelet agents was not available. The lack of these important factors may cause biases in evaluation of CVE.*

[Response to Q2]

Following your comment, we re-analyzed the adjusted model adding statins and antiplatelets as the potential confounders. As the result of re-analysis, we identified primary renal disease (PRD) patients also have the risk for non-renal outcomes. Please, see figures and tables (**Fig. 3** and **4**, **S1** and **S2 Table**). Also we modified the manuscript.

*Minor issue:Q3: What is the definition of hyperuricemia? What is the cut-point value of hyperuricemia used in the current study? Was the hyperuricemia defined according to the initial single laboratory data or the average of serial blood exams during follow-up? Were patients divided into quartiles according to serum UA level? The authors should define hyperuricemia more clearly.*

[Response to Q3]

First of all, hyperuricemia in this report was defined the serum uric acid level of 7.0 mg/dL or higher regardless of sex. Regarding this point, we added the following comment in Methods section.

Line 91, Hyperuricemia was defined the serum UA level of 7.0 mg/dL or higher regardless of sex.

And we demonstrate 1-mg/dL increase in uric acid level is associated with a hazard ratio of 1.250 [1.035 – 1.510] for non-renal outcome in hypertensive renal disease patients. In this analysis, we used uric acid level at the time of enrollment as continuous variable. And we detected that UA 6.0 mg/dL in PRD and UA 6.8 mg/dL in HTN are optimal cut off value from ROC analysis. Regarding this point, we added the following comment in Result section.

Line 174-176, UA 6.0 mg/dL in PRD and UA 6.8 mg/dL in HTN are optimal cut off value from ROC analysis. AUC of ROC curve and p-value are 0.611 (95%CI, 0.537-0.685, p=0.004) in PRD and 0.658 (95%CI, 0.584-0.732, p=0.012) in HTN.

*Q4: How to define medication use? Did a single prescription of medication fit the definition of medication use? Or a continuous use of a drug for a period of time could be regarded as medication use. The authors should define medication use more clearly.*

[Response to Q4]

Patients who have continued to use allopurinol at the time of entry were registered as medication users. Therefore, inclusion criteria as drug user are not set by the length of the administration period. Regarding this point, we added the following comment in Methods section.

Line 88-90, Patients who have continued to use each drug at the time of entry are registered as medication users, and inclusion criteria as drug user are not set by the length of the administration period.

*Q5: In the current study, allopurinol did not decrease the risks for renal and non-renal outcomes. However, information about the use of other UA lowering agents, such as febuxostat and benzbromarone, was not available. That may lead to biases when evaluating the effects of UA lowering agent and clinical outcomes. Is it possible to consider all the UA lowering agents in the current study?*

[Response to Q5]

In the current study, UA level and allopurinol users are extracted and analyzed only at the time of entry (from 2006 to 2008). And very few patients were using uric acid lowering agents other than allopurinol. For this reason, febuxostat, which has been available since 2011 in Japan, or benzbromarone are not considered in this analysis. Of course, it is expected that there are not a few cases in which allopurinol was switched to febuxostat or topiroxostat during the course of treatment, and possibility of bias is considered, so this point was added to the limitation.

Line 240-242, Second, details of the adjustment of allopurinol, such as discontinuation, reduction, and increase, were not available, also, the data of switching to other XOR inhibitors, such as febuxostat or topiroxostat in the treatment course is lacking.

*Q6: The title of the present study “ Relationships of underlying disease causing chronic kidney disease to hyperuricemia and clinical outcomes “ was a little bit confusing. Could the authors make it more clear?*

[Response to Q6]

We changed the title of the present study from “Relationships of underlying disease causing chronic kidney disease to hyperuricemia and clinical outcomes” to “Different clinical impact of hyperuricemia according to etiologies of chronic kidney disease: Gonryo Study”.
