# Supplementary material for: Different clinical impact of hyperuricemia according to etiologies of chronic kidney disease: Gonryo Study
Source: PLoS One. 2021 Mar 25;16(3):e0249240. doi: 10.1371/journal.pone.0249240 (PMC7993817; doi:10.1371/journal.pone.0249240)
Supplement: Attachment — Submitted filename: Response to Reviewers R1.docx [file pone.0249240.s004.docx]

**Reviewer #1**: *(No Response)*

**Reviewer #2**: *Q1: The authors added the definition of HTN and DN in the revised manuscript. However, the authors did not mention about how to classify if a patient had both diabetes mellitus and hypertension simultaneously. As shown in Table 1, nearly one-third patients in the HTN group had diabetes mellitus. Why were these patients regarded as HTN rather than DN? Since low biopsy rate in patients from HTN and DN groups, more convincing and detailed classification criteria to differentiate HTN from DN should be described. Otherwise, the authors should mention that misclassification is possible and the data should be interpreted with caution in the limitation section.*

[Response]
Patients who have not had renal biopsy and who have both hypertension and diabetes at the same time were classified as either HTN or DN by the attending physicians’ judgment based on the medical history, urine test and presence or absence of diabetic retinopathy. Hypertensive nephropathy was defined by preceding history of hypertension with absence of other possible disorders, including cases with biopsy findings of nephrosclerosis, and diabetic nephropathy was defined by preceding history of diabetes accompanying nephropathy with absence of other possible renal disorders, including cases with biopsy findings of diabetic nephropathy or those who presenting nephropathy with diabetic retinopathy in the absence of other possible disorders. However, biopsy rate is low in HTN and DN. And it is considered difficult to distinguish between HTN and DN in a strict sense without biopsy finding. We think this point is considered as the limitation of this study, so we added the following comments.

Line 247-250 (Discussion) ~~Third, renal biopsy was performed in 50.6% of all patients, and only 12.8% of hypertensive renal disease patients.~~ Third, renal biopsy was performed only 12.8% in HTN group, and 17.1% in DN group, and nearly one-third patients in the HTN group had diabetes mellitus. It is considered difficult to distinguish between HTN and DN in a strict sense without biopsy. So, caution should be taken in interpreting the data.

*Q2: Since the authors analyzed UA as a continuous variable, and the relationship between UA and clinical outcomes was demonstrated as hazard ratios per 1 mg/dl greater UA level, they should mention this clearly in the Methods section, and described the results more precisely in the manuscript (Abstract, Results section, Discuss section, and caption of figures)*

[Response]

We modified the manuscript about description for relationship between UA and clinical outcomes as follows.

Line 42-43, (Abstract) ~~on multivariate Cox proportional analysis (adjusted hazard ratio [95% confidence interval] PRD, 1.248 [1.003 – 1.553]; HTN, 1.250 [1.035 – 1.510]).~~ Per 1 mg/dl higher UA level, multivariable adjusted hazard ratio was 1.248 (95% CI: 1.003 to 1.553) for PRD, and 1.250 (1.035 to 1.510) for HTN.

Line 139-141, (Methods) We analyzed UA as a continuous variable and investigated associations between UA per 1 mg/dl higher value with ESRD, CVEs, and all-cause mortality.

Line 166-167 (Results) risk for ESRD (adjusted HR per 1 mg/dl higher UA level was 1.101, [95%CI 1.009-1.202], p=0.031)

Line 174-177 (Results) In the subgroup analysis, UA was associated with an increased risk for the non-renal outcome in PRD and HTN group. ~~PRD and HTN was associated with an increased risk for the non-renal outcome (adjusted HR [95% Cl] PRD, 1.248 [1.003 – 1.553]; HTN, 1.250 [1.035 – 1.510])~~ Per 1 mg/dl higher UA level, multivariable adjusted HR was 1.248 (95% CI: 1.003 to 1.553) for PRD, and 1.250 (1.035 to 1.510) for HTN.

Line 192-194 (Discussion) We analyzed UA as a continuous variable and demonstrated that per 1 mg/dl higher UA level, multivariable adjusted HR was 1.248 (95% CI: 1.003 to 1.553) for PRD, and 1.250 (1.035 to 1.510) for HTN.

Legends for Figure 3, ~~Adjusted for underlying disease of CKD, eGFR, urinary protein, systolic blood pressure, past history of cardiac disease, ACEis/ARBs, diuretics, statins, antiplatelets, allopurinol, age, sex, smoking, body mass index, hemoglobin, albumin.~~ Associations are measured per 1 mg/dl increment of UA using Cox proportional hazard regression. Hazard ratios (HRs) were adjusted for underlying disease of CKD, eGFR, urinary protein, systolic blood pressure, past history of cardiac disease, ACEi/ARBs, diuretics, statins, antiplatelets, allopurinol, age, sex, smoking, body mass index, hemoglobin, albumin.

Legends for Figure 4, ~~Adjusted for underlying disease of CKD, eGFR, urinary protein, systolic blood pressure, past history of cardiac disease, ACEis/ARBs, diuretics, statins, antiplatelets, allopurinol, age, sex, smoking, body mass index, hemoglobin, albumin.~~ Associations are measured per 1 mg/dl increment of UA using Cox proportional hazard regression. Hazard ratios (HRs) were adjusted for underlying disease of CKD, eGFR, urinary protein, systolic blood pressure, past history of cardiac disease, ACEi/ARBs, diuretics, statins, antiplatelets, allopurinol, age, sex, smoking, body mass index, hemoglobin, albumin.

*Q3: The authors re-analyzed the HR after taking statin and anti-platelet into consideration in the revised manuscript. In the Statistical analysis sections and Results section, adjusting for statin and anti-platelet should be mentioned. Please make sure that all corresponding description in the manuscript have been modified.*

[Response]

We checked all corresponding description in the manuscript and modified as follows.

Line 142-145 (Methods) Analyzed variables included underlying disease of CKD, eGFR, urinary protein, systolic blood pressure, past history of cardiac disease, ACE inhibitor and/or ARB use, diuretic use, statins, antiplatelets, age, sex, smoking habits, BMI, hemoglobin, and albumin.

Line 162-166 (Results) In all subjects, UA was not an independent risk factor for ESRD with the Cox proportional hazard model adjusted for underlying disease causing CKD, eGFR, urinary protein, systolic blood pressure, past history of cardiac disease, ACE inhibitors/ARBs, diuretics, statins, antiplatelets, age, sex, smoking, BMI, hemoglobin, and albumin.

Line 171-174 (Results) In all subjects, UA was not an independent risk factor for the non-renal outcome with the Cox proportional hazard model adjusted for underlying disease causing CKD, eGFR, urinary protein, systolic blood pressure, past history of cardiac disease, ACE inhibitors/ARBs, diuretics, statins, antiplatelets, age, sex, smoking, BMI, hemoglobin, and albumin.

*Q4: In line 172-173, The sentence "In the subgroup analysis, PRD and HTN was associated with an increased risk for the non-renal outcome" was confusing. Please make it more clear*.

[Response]

We modified the sentence as follows according to the suggestion.

Line 174-176, In the subgroup analysis, ~~PRD and HTN was associated with an increased risk for the non-renal outcome~~ UA was associated with an increased risk for the non-renal outcome in PRD and HTN group
